# Entomological journals and publishing in Japan

**DOI:** 10.1007/s13355-015-0386-z

**Published:** 2016-01-11

**Authors:** Takema Fukatsu

**Affiliations:** Bioproduction Research Institute, National Institute of Advanced Industrial Science and Technology (AIST), 1-1-1 Higashi, Tsukuba, 305-8566 Japan; Department of Biological Sciences, Graduate School of Science, The University of Tokyo, Tokyo, 113-0033 Japan; Graduate School of Life and Environmental Sciences, University of Tsukuba, Tsukuba, 305-8572 Japan

**Keywords:** Entomology, Journal, Japan, Open-access, J-stage, CiNii, The Union of Japanese Societies for Insect Sciences, The Union of the Japanese Societies for Systematic Biology, The Union of Japanese Societies for Natural History, The Union of Japanese Societies for Biological Science

## Abstract

Here I present an overview of entomological journals and publishing in Japan, thereby providing a convenient portal to the valuable scientific resources for the world’s entomological researchers and scientific communities. Currently, except for several international journals published fully in English such as *Applied Entomology and Zoology* and *Entomological Science*, many entomological and entomology-related journals in Japan are not indexed by major scientific databases like Web of Science, and therefore they are neither conveniently recognizable nor accessible for the world’s entomological communities. However, I point out that many of the contents of such journals are freely available via Japan’s public platforms for electronic scientific literature, Japan Science and Technology Information Aggregator, Electronic (J-stage) or Citation Information by National Institute of Informatics (CiNii). Here I list 32 entomological and entomology-related societies and their 45 journals, the majority of which belong to either the Union of Japanese Societies for Insect Sciences (UJSIS), the Union of the Japanese Societies for Systematic Biology (UJSSB), the Union of Japanese Societies for Natural History (UJSNH), or the Union of Japanese Societies for Biological Science (UJSBS), with their respective URL and open-access availability.

## Introduction

Entomology, the scientific study of insects, is of general importance with both basic and applied aspects (Gullan and Cranston 2014). Although human interest in insects has a long history dating back to the prehistoric era, entomology as a scientific discipline emerged in the sixteenth century and was established by the eighteenth century (Smith 1973). Currently, a number of entomological journals are publishing numerous scientific articles of entomological and entomology-related research fields all over the world. For example, the Journal Citation Reports 2014 (http://about.jcr.incites.thomsonreuters.com) is indexing 92 journals under the category of “Entomology”, although the number no doubt represents merely the tip of an iceberg.

In Japan, although some pioneering descriptive studies have been conducted under the scheme of herbalism and pharmacognosy since the seventeenth century, entomology in a modern style was introduced as a part of zoology and launched after the opening of Japan to the world upon the Meiji Restoration from 1868 and after (Hayashi 2004). Here, it should also be noted that Japanese people have traditionally loved and highly appreciated insects, which are deeply interwoven into their cultural and mental backgrounds. Even in the twenty-first century, many Japanese people enjoy watching fireflies glittering over flowing streams in summer (Oba et al. 2011), listening to sweet notes of crickets, grasshoppers, and cicadas (Ryan 1996), and collecting, rearing, and trading spectacular rhino and stag beetles (Brock 2006; Goka et al. 2004). Concordantly, a number of entomological journals, proceedings, reports, newsletters, and books are published not only by academic societies but also by agricultural and other biological research institutes and local stations, natural history museums, amateur entomologist associations, etc.

Unfortunately, the wealth of entomological knowledge accumulated in Japan is not necessarily easily accessible by the world’s entomological researchers and scientific communities, primarily because the majority of the literature is written in Japanese, and secondarily because the majority of the articles are not indexed by major online searching platforms for scientific literature such as Web of Science and PubMed. However, I point out that, although not widely recognized outside Japan, some, if not all, contents of such journals are freely available via Japan’s public open-access platforms, J-stage or CiNii (see “Box 1”).

In this article, I introduce the principal entomological and entomology-related academic societies of Japan and their respective journals, URLs, and open-access availability, thereby providing a convenient portal to the valuable scientific resources for the world’s entomological researchers and scientific communities. Note that all the information described here is valid as of December 2015, which will be subject to change in the future.

## Entomological and entomology-related journals in Japan

It is virtually impossible to cover all entomological journals, proceedings, reports, newsletters, and other literature published in Japan. Here I highlight 24 entomological and entomology-related academic societies, which are members of UJSIS, UJSSB, UJSNH and/or UJSBS (see “Box 2”) and publishing a total of 37 scientific journals. These societies are selected because of (1) continuous and active functioning as academic societies, (2) authorization by the esteemed academic unions, (3) publishing academic journals in English and/or in Japanese with an English summary, (4) online accessibility via the website of the societies, and (5) online searchability and/or open-accessibility of the journals’ contents. Furthermore, six proceedings/annual reports of local plant protection societies of Japan are listed, which publish many entomology-related papers that are open-access with an English title and/or summary. In addition, two entomology-specialized periodicals, *Insecta Matsumurana* from Hokkaido University and *Esakia* from Kyushu University, are introduced. In total, 32 entomological and entomology-related societies and their 45 journals are highlighted in this article (Table 1).

**Table 1:** Insect-related academic societies of Japan and their scientific journals (information is valid as of December 2015)

Society name^a^ URL^b^	Language^e^	2015 Vol. (No.)^f^	ISSN	Current publisher
Journal name [proportion of entomological papers in 2015]^b,c^ URLs^b,c,d^				
Full Entomological Journals^g^				
The Japanese Society of Applied Entomology and Zoology^IS^ http://en.odokon.org				
*Applied Entomology and Zoology* [65/67 = 97 %] http://www.springer.com/life+sciences/entomology/journal/13355 https://www.jstage.jst.go.jp/browse/aez (OA 1966–2010)	E	50 (4)	0003-6862	Springer
*Japanese Journal of Applied Entomology and Zoology* [22/22 = 100 %] http://odokon.org/en/aez_ja.php https://www.jstage.jst.go.jp/browse/jjaez/-char/en/ (OA 2000–latest) http://ci.nii.ac.jp/vol_issue/nels/AN00186121_en.html (OA 1957–2009)	J (E)	59 (4)	0021-4914	Jpn Soc Appl Entomol Zool
The Entomological Society of Japan^IS,SB,NH^ http://www.entsoc.jp/e/e-home.htm				
*Entomological Science* [63/63 = 100 %] http://onlinelibrary.wiley.com/journal/10.1111/(ISSN)1479-8298	E	18 (4)	1479-8298	Wiley
*Japanese Journal of Entomology* (*New Series*) [18/18 = 100 %] http://www.entsoc.jp/e/e-9.htm http://ci.nii.ac.jp/vol_issue/nels/AA11248127_en.html (OA 1998–2013)	J (E’)	18 (4)	1343-8794	Entomol Soc Jpn
The Japan Society of Medical Entomology and Zoology^IS,NH ^ http://jsmez.gr.jp/wordpress/				
*Medical Entomology and Zoology* [19/19 = 100 %] http://jsmez.gr.jp/wordpress/journal https://www.jstage.jst.go.jp/browse/mez (OA 2010–latest) http://ci.nii.ac.jp/vol_issue/nels/AN00021948_en.html (OA 1950–2010)	E J (E)	66 (4)	0424-7086	Jpn Soc Med Entomol Zool
The Japanese Society of Sericultural Science^IS ^ http://www.jsss.or.jp ^J^				
*Journal of Insect Biotechnology and Sericology* [9/9 = 100 %] http://jsss.or.jp/modules/pico3/index.php?content_id=1 ^J^ https://www.jstage.jst.go.jp/browse/jibs (OA 2001–latest) https://www.jstage.jst.go.jp/browse/kontyushigen (OA 1930–2005)	E	84 (3)	1346-8073	Jpn Soc Sericultural Sci
*Sanshi*-*Konchu Biotech* [19/19 = 100 %] http://jsss.or.jp/modules/pico3/index.php?content_id=1 ^J^ https://www.jstage.jst.go.jp/browse/konchubiotec/-char/ja/ ^J^ (OA 2006–latest) https://www.jstage.jst.go.jp/browse/kontyushigen/-char/ja/ ^J^ (OA 1930–2005)	J	84 (3)	1881-0551	Jpn Soc Sericultural Sci
The Japanese Society for Wild Silkmoths^IS ^ http://jsws.web.fc2.com ^J^				
*International Journal of Wild Silkmoth and Silk* ^h^ [6/7 = 86 %] http://jswsmo.appspot.com/IntJourConte.html	E J (E)	19 (1)	1340-4725	Jpn Soc Wild Silkmoths
*Yasan (Wild Silkworm News)* ^h^ [7/7 = 100 %] http://jswsmo.appspot.com/kaihou2.html ^J^	J	75–76	0913-0772	Jpn Soc Wild Silkmoths
The Lepidopterological Society of Japan^IS,NH ^ http://www.lepi-jp.org/index_e.htm				
*Lepidoptera Science* [18/18 = 100 %] http://www.lepi-jp.org/index_e_publication.htm http://ci.nii.ac.jp/vol_issue/nels/AN00147618_en.html (OA 1949–2013)	E J (E)	66 (4)	0024-0974	Lepidopterol Soc Jpn
*Yadoriga* [38/40 = 95 %] http://www.lepi-jp.org/index_e_publication.htm http://ci.nii.ac.jp/vol_issue/nels/AN00242506_en.html (OA 1955–2013)	J	244–247	0513-417X	Lepidopterol Soc Jpn
Coleopterological Society of Japan^SB ^ http://kochugakkai.sakura.ne.jp/English/index2.html				
*Elytra (New Series)* [74/74 = 100 %] http://kochugakkai.sakura.ne.jp/publication/elytra/elytra-bibliography.html	E	5 (2)	2185-6885	Coleopterol Soc Jpn
*Sayabane (New Series)* [70/70 = 100 %] http://kochugakkai.sakura.ne.jp/publication/sayabane/sayabane-bibliography.html ^J^	J (E’)	17–20	2185-9787	Coleopterol Soc Jpn
Arachnological Society of Japan^IS,SB,NH ^ http://www.arachnology.jp/index-e.php				
*Acta Arachnologica* [16/16 = 100 %] http://www.arachnology.jp/AA-e.php?n=6 https://www.jstage.jst.go.jp/browse/asjaa (OA 1936–latest)	E J (E’)	64 (2)	0001-5202	Arachnol Soc Jpn
The Acarological Society of Japan^IS,SB ^ http://en.acarology-japan.org				
*Journal of the Acarological Society of Japan* [5/5 = 100 %] http://en.acarology-japan.org/?page_id=16 https://www.jstage.jst.go.jp/browse/acari (OA 1992-latest)	E J (E)	24 (2)	0918-1067	Acarol Soc Jpn
Japanese Society of Systematic Entomology^NH ^ https://sites.google.com/site/jjsystent/ ^J^				
*Japanese Journal of Systematic Entomology* [25/25 = 100 %] https://sites.google.com/site/jjsystent/old-volume/contents	E	21 (2)	1341-1160	Jpn Soc Syst Entomol
Biological Journals Publishing Entomological Papers				
The Zoological Society of Japan^IS,NH,BS ^ http://www.zoology.or.jp/en/				
*Zoological Letters* [8/35 = 23 %] http://zoologicalletters.biomedcentral.com (OA 2015-latest)	E	1 (–)	2056-306X	BioMed Central
*Zoological Science* [16/78 = 21 %] http://www.bioone.org/loi/jzoo (OA 1995–2004)	E	32 (6)	0289-0003	Zool Soc Jpn
The Japanese Society of Systematic Zoology^SB,NH ^ http://jssz.sakura.ne.jp/index-e.html				
*Species Diversity* [0/23 = 0 %] http://jssz.sakura.ne.jp/spdiv/index.html (OA 2008-latest) http://ci.nii.ac.jp/vol_issue/nels/AA11114903_en.html (OA 1996–2012)	E	20 (2)	1342-1670	Jpn Soc Syst Zool
*Taxa* [0/11 = 0 %] http://jssz.sakura.ne.jp/taxa/index.html ^J^ http://ci.nii.ac.jp/vol_issue/nels/AN1056362X_en.html (OA 1996–2012)	J (E)	38–39	1342-2367	Jpn Soc Syst Zool
The Japanese Society of Soil Zoology^SB ^ http://soilzoology.jp/en/				
*Edaphologia* [4/7 = 57 %] http://soilzoology.jp/en/publications/ http://ci.nii.ac.jp/vol_issue/nels/AN00334874_en.html (OA 2001–2014)	E J (E)	96–97	0389-1445	Jpn Soc Soil Zool
The Japanese Nematological Society^IS,SB ^ http://senchug.org/e/index-e.html				
*Nematological Research* [0/13 = 0 %] https://www.jstage.jst.go.jp/browse/jjn (OA 1993–2014) https://www.jstage.jst.go.jp/browse/jjn1972 (OA 1972–1992)	E J (E’)	45 (2)	0919-6765	Jpn Nematol Soc
Japan Ethological Society^NH ^ http://www.ethology.jp/index-e.html				
*Journal of Ethology* [4/27 = 15 %] http://www.ethology.jp/JE-e.html http://link.springer.com/journal/volumesAndIssues/10164	E	33 (3)	0289-0771	Springer
The Ecological Society of Japan^NH,BS ^ http://www.esj.ne.jp/esj/e_index.html				
*Ecological Research* [11/97 = 11 %] http://www.esj.ne.jp/er/index.html http://link.springer.com/journal/volumesAndIssues/11284	E	30 (6)	0912-3814	Springer
*Japanese Journal of Ecology* [5/30 = 17 %] http://www.esj.ne.jp/esj/JJE/index.html ^J^ http://ci.nii.ac.jp/vol_issue/nels/AN00193852_en.html (OA 1954–latest)	J (E)	65 (3)	0021-5007	Ecol Soc Jpn
*Japanese Journal of Conservation Ecology* [2/16 = 13 %] http://www.esj.ne.jp/esj/JJCE/index.html ^J^ http://ci.nii.ac.jp/vol_issue/nels/AA11857952_en.html (OA 1996–2013)	J (E)	20 (2)	1342-4327	Ecol Soc Jpn
The Biogeographical Society of Japan^NH ^ http://biogeo.a.la9.jp ^J^				
*Biogeography* [2/19 = 11 %] http://biogeo.a.la9.jp ^J^	E	17 (1)	1345-0662	Biogeographic Soc Jpn
*Bulletin of the Biogeographical Society of Japan* [3/35 = 9 %] http://biogeo.a.la9.jp ^J^	J (E)	70 (1)	0067-8716	Biogeographic Soc Jpn
The Society of Population Ecology^BS ^ http://www.populationecology.org				
*Population Ecology* [13/57 = 23 %] http://link.springer.com/journal/volumesAndIssues/10144	E	57 (4)	1438-3896	Springer
The Japanese Society for Comparative Physiology and Biochemistry^IS,BS ^ http://jscpb.org/ja/23395/				
*Comparative Physiology and Biochemistry* [5/11 = 45 %] https://www.jstage.jst.go.jp/browse/hikakuseiriseika (OA 1990–latest) https://www.jstage.jst.go.jp/browse/hikakuseiriseika1984 (OA 1984–1989)	J (E)	32 (4)	0916-3786	Jpn Soc Comp Physiol Biochem
The Japan Society for Bioscience, Biotechnology, and Agrochemistry^IS ^ http://www.jsbba.or.jp/e/				
*Bioscience, Biotechnology, and Biochemistry* [2/289 = 1 %] http://www.tandfonline.com/loi/tbbb20#.VhJ0YMtrgu4 https://www.jstage.jst.go.jp/browse/bbb (OA 1992–2013) https://www.jstage.jst.go.jp/browse/bbb1961 (OA 1961–1991) https://www.jstage.jst.go.jp/browse/bbb1924 (OA 1924–1960)	E	79 (12)	0916-8451	Taylor and Francis
*Kagaku To Seibutsu* [8/156 = 5 %] https://katosei.jsbba.or.jp/back_issue.php?btn1_move=on ^J^ https://www.jstage.jst.go.jp/browse/kagakutoseibutsu (OA 1962–2014)	J	53 (12)	0453-073X	Jpn Soc Biosci Biotechnol Agrochem
Pesticide Science Society of Japan^IS ^ http://pssj2.jp/eng/index.html				
*Journal of Pesticide Science* [10/32 = 31 %] http://pssj2.jp/eng/journal/jps.html https://www.jstage.jst.go.jp/browse/jpestics (OA 1975–latest)	E	40 (4)	1348-589X	Pesticide Sci Soc Jpn
*Japanese of Journal of Pesticide Science* [5/23 = 22 %] http://pssj2.jp/eng/journal/ejjps.html https://www.jstage.jst.go.jp/browse/jjpestics/ (OA 2013–latest)	J (E’)	40 (2)	2187-0365	Pesticide Sci Soc Jpn
The Society of Urban Pest Management, Japan^IS ^ http://upm.urbanpest.net ^J^				
*Urban Pest Management* [14/15 = 93 %] http://ci.nii.ac.jp/vol_issue/nels/AA12534176_en.html (OA 2011–2014)	E J (E’)	4 (2)	2186-1498	Soc Urban Pest Manag Jpn
Japanese Society of Environmental Entomology and Zoology^IS ^ http://kandoukon.org/english/english.html				
*Japanese Journal of Environmental Entomology and Zoology* [11/15 = 73 %] http://kandoukon.org/paper/paper.html ^J^ (OA 1989–latest) http://ci.nii.ac.jp/vol_issue/nels/AN10186037_en.html (OA 2006–2008) https://www.jstage.jst.go.jp/browse/jjeez (OA 2004)	J (E)	26 (4)	0915-4698	Jpn Soc Environ Entomol Zool
The Japanese Society of Pestology^IS ^ http://www.pestology.jp ^J^				
*Pestology* [10/10 = 100 %] http://www.pestology.jp/treatise.html ^J^ http://ci.nii.ac.jp/vol_issue/nels/AA12402244_en.html (OA 2005–2014)	J (E’)	30 (2)	1880-3415	Jpn Soc Pestology
Proceedings/Annual Reports of Local Plant Protection Societies of Japan				
The Society of Plant Protection of North Japan http://sppnj.sakura.ne.jp/index.html ^J^				
*Annual Report of the Society of Plant Protection of North Japan* [13/35 = 37 %] http://sppnj.sakura.ne.jp/contents.html#english ^J^ http://agriknowledge.affrc.go.jp/browse/journal/#-//ZZ00015089 ^J ^(OA 1970–2010) https://www.jstage.jst.go.jp/browse/kitanihon1966 (OA 1966–2008) https://www.jstage.jst.go.jp/browse/kitanihon1951 (OA 1951–1965)	J (E’)	66	0368-623X	Soc Plant Prot North Jpn
The Kanto-Tosan Plant Protection Society http://www.ktpps.org/ ^J^				
*Annual Report of the Kanto*-*Tosan Plant Protection Society* [15/40 = 38 %] http://www.ktpps.org/journal.php ^J^ https://www.jstage.jst.go.jp/browse/ktpps (OA 1999–2014) https://www.jstage.jst.go.jp/browse/ktpps1954 (OA 1954–1998)	J (E’)	62	1347-1899	Kanto-Tosan Plant Prot Soc
The Association for Plant Protection of Hokuriku http://hokuriku-byochu.sakura.ne.jp/apph/ ^J^				
*Proceedings of the Association of Plant Protection of Hokuriku* [4/7 = 57 %] http://hokuriku-byochu.sakura.ne.jp/apph/proc.html ^J^ (OA 1995–2014)	J (E’)	64	0388-8053	Assoc Plant Prot Hokuriku
The Kansai Plant Protection Society http://kanbyochu.jpn.org ^J^				
*Annual Report of the Kansai Plant Protection Society* [15/29 = 52 %] http://kanbyochu.jpn.org/?page_id=92 ^J^ https://www.jstage.jst.go.jp/browse/kapps (OA 1958–latest)	E J (E)	57	0387-1002	Kansai Plant Prot Soc
The Association for Plant Protection of Shikoku http://shikoku-shokubo.org ^J^				
*Proceedings of the Association for Plant Protection of Shikoku* [0/5 = 0 %]^h^ http://shikoku-shokubo.org/shikoku-shokubo/htm/show_unit.cgi?mode=category&category=BACKNUMBER ^J^ (OA 1966–2012)	J (E’)	49	0386-0515	Assoc Plant Prot Shikoku
The Association for Plant Protection of Kyushu http://9byochu.sakura.ne.jp/main.html ^J^				
*Kyushu Plant Protection Research* [5/12 = 42 %] http://9byochu.sakura.ne.jp/backNO.html ^J^ https://www.jstage.jst.go.jp/browse/kyubyochu (OA 1955–2013)	J (E’)	61	0385-6410	Assoc Plant Prot Kyushu
Entomological Periodicals from University Departments				
Graduate School of Agriculture, Hokkaido University http://insect3.agr.hokudai.ac.jp/sefu.html				
*Insecta Matsumurana* [3/3 = 100 %] http://insect3.agr.hokudai.ac.jp/insecta-matsumurana/ http://eprints.lib.hokudai.ac.jp/journals/index.php?jname=188 (OA 1926–latest)	E	71	0020-1804	Grad Sch Agr Hokkaido Univ
Entomological Laboratory, Faculty of Agriculture, Kyushu University http://www.agr.kyushu-u.ac.jp/lab/entomology/en/index.html				
*Esakia* [10/10 = 100 %]^h^ https://www.lib.kyushu-u.ac.jp/en/publications_kyushu/esakia (OA 1960–2014)	E	–^i^	0071-1268	Entomol Lab Fac Agr Kyushu Univ

^a^ *IS* UJSIS, *SB* UJSSB, *NH* UJSNH, *BS* UJSBS

^b^ *J* Japanese website only

^c^The latest journal name is shown, although the journal name and website may have changed in the past. For tracking the history, follow the URLs

^d^For example, (OA 1966–2010) means contents of the journal from 1996 to 2010 are open-access via the URL

^e^E, papers in English; J, papers in Japanese without English summary; J (E), papers in Japanese with English summary; J (E’), papers in Japanese optionally with English summary

^f^For example, 50 (4) indicates that the journal published 4 issues in 2015 as volume 50; 75–76 indicates that the journal published 2 issues from No. 75 to No. 76 in 2015 without a volume system; 1 (–) indicates that the journal published papers as volume 1 in 2015 without an issue system; 66 indicates that the journal published a single issue no. 66 in 2015

^g^Defined as journals specialized for publishing papers on insects and other terrestrial arthropods

^h^Since the values for 2015 are not available, the values for 2014 are shown

^i^Publication of the journal was suspended from 2015

## Societies publishing English and Japanese journals

Many, if not all, of the academic societies in Japan are publishing two journals: one is English-based for the international audience while the other is Japanese-based for the domestic audience. The English-based journals range from those that accept papers in English only, journals that mainly publish papers in English but also accept papers in Japanese, and journals that are open to papers both in English and Japanese. In these journals, papers in Japanese almost always accompany English summary. The Japanese-based journals mostly publish papers in Japanese only and the availability of an English summary depends on the journals’ policy. As shown in Table 1, the Japanese Society of Applied Entomology and Zoology, the Entomological Society of Japan, the Japanese Society of Sericultural Science, the Japanese Society for Wild Silkmoths, the Lepidopterological Society of Japan, Coleopterological Society of Japan, the Japanese Society of Systematic Zoology, the Biogeographical Society of Japan, the Japan Society for Bioscience, Biotechnology, and Agrochemistry, and Pesticide Science Society of Japan represent this type of academic society.

The Ecological Society of Japan publishes an English-based journal, *Ecological Research*, and two Japanese-based journals, *Japanese Journal of Ecology* and *Japanese Journal of Conservation Ecology*, which reflect the importance of both basic and applied aspects of ecology in the contemporary world.

## Societies publishing a single journal

Some academic societies in Japan are publishing a single journal. As shown in Table 1, the Japan Ethological Society, the Society of Population Ecology, and the Japanese Society of Systematic Entomology publish a journal fully in English; the Japan Society of Medical Entomology and Zoology, the Arachnological Society of Japan, the Acarological Society of Japan, and the Japanese Nematological Society have a journal publishing papers both in English and Japanese; the Japanese Society for Comparative Physiology and Biochemistry, the Society of Urban Pest Management, Japan, the Japanese Society of Environmental Entomology and Zoology, and the Japanese Society of Pestology have a journal publishing papers substantially in Japanese only and the availability of an English summary depends on the journals’ policy.

## A society publishing dual English journals

Exceptionally, the Zoological Society of Japan is publishing two journals fully in English. In addition to *Zoological Science* that has been published for decades under the BioOne platform, the society launched *Zoological Letters* in 2015 as a new full open-access journal from BioMed Central, thereby intending the rapid handling and seamless publication of a variety of papers in the field of zoology. The new journal places emphasis on topical zoological issues such as the discovery of the sixth digit of frogs (Hayashi et al. 2015), incapability of smelling and tasting revealed by whale genomics (Kishida et al. 2015), description of enigmatic animal phyla (Nakano 2015; Yamasaki et al. 2015), etc., and also actively publishes entomological studies: eight of 35 articles published in 2015 are on insect-related topics including circadian rhythms and clocks (Komada et al. 2015; Numata et al. 2015), ecdysteroid fucntions (Uryu et al. 2015), neural mechanisms for learning (Mizunami et al. 2015), chemical and structural basis of body color (Stavenga et al. 2015), ecological impact of urbanization (Moriyama and Numata 2015), flight muscle structure (Iwamoto 2015), and symbiotic microorganisms (Hosokawa et al. 2015).

## Proceedings/annual reports of local plant protection societies of Japan

In Japan, there are six major local plant protection societies that have been active for decades: the Society of Plant Protection of North Japan (northern part of Japan; Hokkaido and Tohoku regions), the Kanto-Tosan Plant Protection Society (central mainland areas around Kanto-Tokyo), the Association for Plant Protection of Hokuriku (northern coastal mainland Hokuriku areas), the Kansai Plant Protection Society (central mainland areas around Kansai-Osaka), the Association for Plant Protection of Shikoku (southwestern part of Japan; Shikoku region), and the Association for Plant Protection of Kyushu (southwestern part of Japan; Kyushu and Okinawa regions). These societies have been publishing proceedings or annual reports that compile numerous reports and case studies on agricultural pathogens and pest insects, all of which are published with an English title and some with an English summary. These articles are open-access either via J-stage or the website of the relevant society (Table 1).

### *Insecta Matsumurana* and *Esakia*

Historically, introduction and establishment of modern entomology in Japan have been strongly promoted by Hokkaido University and Kyushu University, where the founding professors, Dr. Shonen Matsumura (1872–1960) at Hokkaido University and Dr. Teizo Esaki (1899–1957) at Kyushu University, played important roles (Kamito 1958; Uchida and Watanabe 1961). These universities are still the centers of systematic entomology in Japan and publishing their own entomological journals, *Insecta Matsumurana* and *Esakia* named after the founders. The contents are freely available via institutional repositories of the universities (Table 1). Regrettably, the publication of *Esakia* was suspended from 2015.

## Open-accessibility of journal contents

Table 1 shows that, importantly, the majority of the entomological and entomology-related journals in Japan are completely or partially open-access: 14 journals via J-stage, 10 journals via CiNii, three journals via J-stage and CiNii, one journal via BioMed Central, one journal via BioOne, and four journals via their own institutional repositories. Some are completely open from the latest issue, while others place 6 months, 1 year, or 2 years of restricted period depending on the journals’ policy.

## Indexing and impact factor

Of the 45 entomological and entomology-related journals, only nine journals are indexed by Web of Science and given an impact factor, and only three journals are indexed by PubMed (Table 2), highlighting that J-stage and CiNii are the essential portals for accessing the majority of the entomological and entomology-related papers published in Japan.

**Table 2:** Indexing by major databases and impact factor of insect-related scientific journals of Japan

Journal	Web of Science	PubMed	Impact factor 2014^a^
Full entomological journals			
*Applied Entomology and Zoology*	+	−	1.143
*Entomological Science*	+	−	1.065
*Japanese Journal of Applied Entomology and Zoology*	+	−	0.196
Biological journals publishing entomological papers			
*Population Ecology*	+	−	1.570
*Ecological Research*	+	−	1.296
*Bioscience, Biotechnology, and Biochemistry*	+	+	1.063
*Journal of Ethology*	+	+	0.970
*Zoological Science*	+	+	0.857
*Journal of Pesticide Science*	+	–	0.722

^a^According to Journal Citation Reports, Thomson Reuters

## Concluding remark

Here I provide a convenient portal to entomological journals and literature published in Japan with their respective URL and open-access availability, which are valuable scientific resources for the world’s entomological researchers and scientific communities. I emphasize that many, if not all, of the journal contents are open-access via the public platforms, J-stage and CiNii, dedicated to scientific literature published in Japan. Notably, the journal archiving service of CiNii will be abolished and integrated into J-stage by March 2017 (http://www.nii.ac.jp/nels_soc/about/schedule/ in Japanese only), when the contents of this article should be updated. I hope that this article will help the world’s entomologists to recognize the wealth of scientific knowledge accumulated in Japan and to make use of it for their research and development.

## Box 1 J-stage and CiNii

J-stage (Japan Science and Technology Information Aggregator Electronic; https://www.jstage.jst.go.jp/browse/) was launched in 1988 by the Japan Science and Technology Agency in order to develop Japan’s scientific and technological research by setting up the hardware and software necessary for electronic journal publishing, thereby enabling the instantaneous dissemination of research results to the world. By taking advantage of the system, user organizations like scientific societies in Japan are able to digitize currently published journals and research papers with ease and at low cost. The digitized contents of J-stage are mostly open-access whereas some are restricted depending on the journals’ policy. As of December 2015, J-stage has compiled over 2.6 million articles.

CiNii (Citation Information by National Institute of Informatics; https://support.nii.ac.jp/en/cinii/cinii_outline) was launched in 2004 by the National Institute of Informatics as a searchable database service for academic information of articles, books, journals, and dissertations. From 2011, CiNii Articles (http://ci.nii.ac.jp/en/) for original papers and CiNii Books (http://ci.nii.ac.jp/books/en/) for books were divided, and in 2015, CiNii Dissertations (http://ci.nii.ac.jp/d/?l=en) was created. Some contents of CiNii are open-access whereas others are restricted depending on the journals’ policy. As of December 2015, CiNii has compiled over 1.7 million articles.

J-stage and CiNii are independent platforms that cover different fields but some overlap. J-stage tends to be directed to scientific, technological, and medical journals, whereas CiNii also covers social scientific fields. Currently, some services of J-stage and CiNii are mutually linked for user convenience. In 2014, the National Institute of Informatics announced that the journal archiving service of CiNii is scheduled to be abolished and integrated into J-stage by March 2017 (http://www.nii.ac.jp/nels_soc/about/ in Japanese only).

## Box 2 UJSIS, UJSSB, UJSNH, and UJSBS

UJSIS (the Union of Japanese Societies for Insect Sciences; http://www.insect-sciences.jp/en/) was established in 2010 in order to promote the advancement of insect-related sciences and the dissemination of the achievements to the general public and society both in Japan and in the world. Currently 17 academic societies related to insect sciences are members of UJSIS, of which 16 societies (except for ICIPE Japan) are publishing one or two periodical scientific journals. Of these, eight societies, the Japanese Society of Applied Entomology and Zoology, the Entomological Society of Japan, the Japan Society of Medical Entomology and Zoology, the Japanese Society of Sericultural Science, the Japanese Society for Wild Silkmoths, the Lepidopterological Society of Japan, Arachnological Society of Japan, and the Acarological Society of Japan, are publishing entomological journals that are dedicated to studies on insects and other terrestrial arthropods, whereas eight societies, the Zoological Society of Japan, the Japanese Nematological Society, the Japanese Society for Comparative Physiology and Biochemistry, the Japan Society for Bioscience, Biotechnology, and Agrochemistry, Pesticide Science Society of Japan, the Society of Urban Pest Management, Japan, Japanese Society of Environmental Entomology and Zoology, and the Japanese Society of Pestology, are publishing more general biological journals that also treat insect-related topics (Table 1).

UJSSB (the Union of the Japanese Societies for Systematic Biology; http://www.ujssb.org in Japanese only) was established in 2002 in order to promote the research and education regarding the taxonomy and systematics of organisms, thereby contributing to development and outreach of the research fields. Currently 25 academic societies are members of UJSSB, of which three societies, the Coleopterological Society of Japan, the Japanese Society of Systematic Zoology, and the Japanese Society of Soil Zoology, are entomology-related and added to the list in addition to the UJSIS members (Table 1).

UJSNH (the Union of the Japanese Societies for Natural History; http://ujsnh.org/index.html in Japanese only) was established in 1995 in order to promote the research and education in scientific fields related to natural history. Currently 40 academic societies are members of UJSNH, of which four societies, the Japanese Society of Systematic Entomology, the Japan Ethological Society, the Ecological Society of Japan, and the Biogeographical Society of Japan, are entomology-related and added to the list in addition to the UJSIS and UJSSB members (Table 1).

UJSBS (the Union of Japanese Societies for Biological Science; http://www.nacos.com/seikaren/ in Japanese only) was established in 1999 in order to facilitate communication and cooperation among academic societies in scientific fields related to biological and life science. Currently 29 academic societies are members of UJSBS, of which the Society of Population Ecology is entomology-related and added to the list in addition to the UJSIS, UJSSB, and UJSNH members (Table 1).
